# Endoscopic resection as a “resect-to-confirm” strategy after neoadjuvant downstaging for remnant gastric cancer

**DOI:** 10.1055/a-2836-1658

**Published:** 2026-04-20

**Authors:** Xinye Zuo, Qianqian Chen, Chen Du, Qun Shao, Jiayan Zhou, Zhikuan Wang, Enqiang Linghu

**Affiliations:** 1 104607 Department of Gastroenterology, Chinese PLA General Hospital, Beijing, China; 2 12538 School of Medicine, Nankai University, Tianjin, China; 3 104607 Medical School of Chinese PLA, Beijing, China; 4 26460 Department of Oncology, The Fifth Medical Center of Chinese PLA General Hospital, Beijing, China

For locally advanced gastric remnant cancer (GRC), radical resection surgery remains the first-line clinical treatment 1 but causes permanent organ loss and functional decline 2 . Recent advances in neoadjuvant therapy (NAT), particularly targeted therapy (anti-CLDN18.2), have markedly elevated pathological complete response rates 3 , enabling exploration of organ-preserving strategies. We report a case of GRC treated with endoscopic local non-full-thickness resection (E-LNFR) after achieving the clinical complete response (cCR) following NAT.

**Fig. 1:**
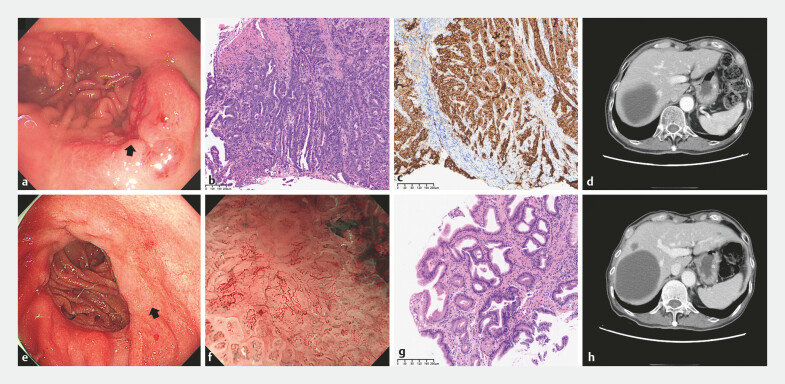
Pre‑therapeutic and post‑neoadjuvant diagnostic evaluations. **a** Anastomotic ulceration under white-light endoscopy (pre‑neoadjuvant; the black arrow indicates the lesion). **b** Pre‑treatment biopsy histopathology showing moderately differentiated adenocarcinoma (×100). **c** Immunohistochemistry demonstrating high Claudin 18.2 expression. **d** CT findings of the lesion prior to neoadjuvant therapy (the white asterisk indicates the lesion). **e** Anastomotic erosion under white light gastroscopy after neoadjuvant therapy (the black arrow indicates the lesion). **f** Magnifying gastroscopy revealed atypical tumor vessels after neoadjuvant therapy. **g** Post-treatment biopsy demonstrating inflammation without neoplasia (×100). **h** CT presentation of the lesion post-neoadjuvant therapy (the white asterisk indicates the lesion). CT, computed tomography.

A 69-year-old man with prior subtotal gastrectomy (24 years ago for duodenal ulcer) presented with an ulcerative lesion on the gastric remnant anastomosis ( Fig. 1 **a** ). Biopsy revealed moderately differentiated adenocarcinoma with high CLDN18.2 expression, HER2(–) ( Fig. 1 **b, c** ). Computed tomography (CT)/positron emission tomography-CT showed stage cT2N0M0 ( Fig. 1 **d** ). The patient declined radical total gastrectomy. After multidisciplinary discussion (MDT), a 3-week NAT regimen was formulated: oxaliplatin (130 mg/m ^2^ , d1) + capecitabine (1,000 mg/m ^2^ , bid, d1–14) + zolbetuximab (800 mg/m ^2^ , d1). The patient completed two cycles but stopped due to grade 3 vomiting (CTCAE v5.0). Follow-up gastroscopy and radiology confirmed tumor regression, supporting the cCR ( Fig. 1 **e–h** ).

**Fig. 2:**
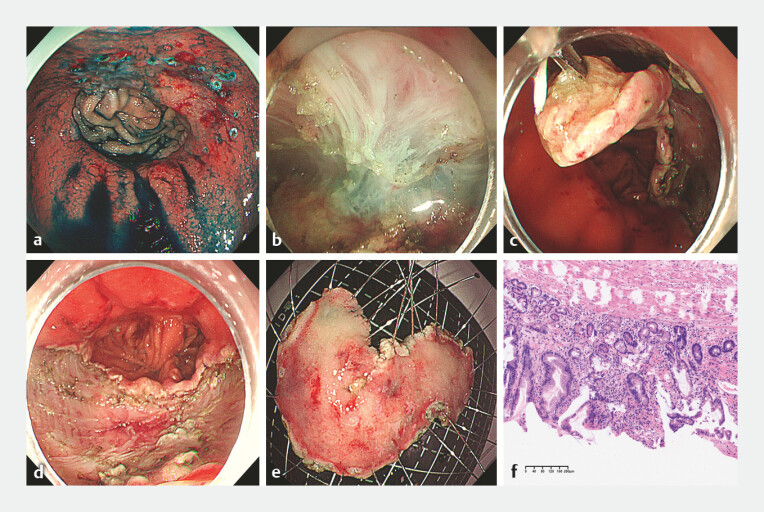
The surgical procedure of E-LNFR. **a** Determination of the resection margin and marking of the lesion under endoscopy. **b** Incision of the mucosal layer and dissection of the submucosal layer. **c** Assistance with dental floss-tissue clip traction to facilitate resection of the anastomotic lesion. **d** Post‑resection wound bed after complete lesion removal. **e** A gross photograph of the resected specimen. **f** Postoperative pathology indicating low-grade dysplasia (×100). E-LNFR, endoscopic local non-full-thickness resection.

To clarify pathological response and achieve curative treatment simultaneously, the MDT decided on E-LNFR ( Video 1 ). First, indigo carmine was sprayed to delineate lesion margins and argon plasma coagulation for boundary marking ( Fig. 2 **a** ). After submucosal injection, mucosal incision and dissection were done on the anastomosis’s oral side ( Fig. 2 **b** ). Dental floss-clip traction was used to expose the anastomotic lesion ( Fig. 2 **c** ). En bloc resection along the anastomosis was completed ( Fig. 2 **d** ). The specimen size was 4.5 × 3.8 cm ( Fig. 2 **e** ). Pathology revealed TRG 0 with negative margins ( Fig. 2 **f** ), confirming the pathological complete response. Postoperative recovery was uneventful.

E-LNFR for gastric remnant cancer. E-LNFR, endoscopic local non-full-thickness resection.Video 1

This case introduces the “resect-to-confirm” strategy – an organ-preserving approach combining pathological verification with curative local resection for NAT-responsive gastric cancer. Prospective studies with a long-term oncologic follow-up are urgently needed.

Endoscopy_UCTN_Code_TTT_1AO_2AG_3AD Endoscopy_UCTN_Code_CCL_1AB_2AD_3AB
